# A Deep Learning Framework for Enhancing High-Frequency Optical Fiber Vibration Sensing from Low-Sampling-Rate FBG Interrogators

**DOI:** 10.3390/s25134047

**Published:** 2025-06-29

**Authors:** Mentari Putri Jati, Cheng-Kai Yao, Yen-Chih Wu, Muhammad Irfan Luthfi, Sung-Ho Yang, Amare Mulatie Dehnaw, Peng-Chun Peng

**Affiliations:** 1 Department of Electro-Optical Engineering, National Taipei University of Technology, Taipei 10608, Taiwan or mentariputrijati@uny.ac.id (M.P.J.); t111659004@ntut.org.tw (C.-K.Y.); t112c52006@ntut.org.tw (Y.-C.W.); t113658061@ntut.edu.tw (S.-H.Y.); amare.mulatie@ntut.edu.tw (A.M.D.); 2 Department of Electrical and Electronics Engineering, Vocational Faculty, Universitas Negeri Yogyakarta, Yogyakarta 55281, Indonesia; 3 Department of Electronics and Informatics Engineering Education, Engineering Faculty, Universitas Negeri, Yogyakarta 55281, Indonesia; m.irfanluthfi@uny.ac.id

**Keywords:** electric motor vibration, fiber sensing, fiber Bragg grating interrogator, deep neural networks

## Abstract

This study introduces a novel deep neural network (DNN) framework tailored to breaking the sampling limit for high-frequency vibration recognition using fiber Bragg grating (FBG) sensors in conjunction with low-power, low-sampling-rate FBG interrogators. These interrogators, while energy-efficient, are inherently limited by constrained acquisition rates, leading to severe undersampling and the obfuscation of fine spectral details essential for accurate vibration analysis. The proposed method circumvents this limitation by operating solely on raw time-domain signals, learning to recognize high-frequency and extremely close vibrational components accurately. Extensive validation using the combination of simulated and experimental datasets demonstrates the model’s superiority in frequency discrimination across a broad vibrational spectrum. This approach is expected to be a significant advancement in intelligent optical vibration sensing and compact, low-power condition monitoring solutions in complex environments.

## 1. Introduction

Vibration sensing based on optical fiber sensors plays a critical role in numerous domains, ranging from industrial fast-rotating machinery monitoring and diagnostics to emerging fields such as smart agriculture, cyber–physical systems, downhole measurement, energy harvesting, and autonomous vehicle systems [1,2,3,4,5,6]. These applications that produce high-frequency waveforms increasingly demand high-sampling-resolution vibration analysis to ensure operational integrity, predictive maintenance, and system-level intelligence.

The acquisition of high-frequenacy vibrational phenomena in optical fiber sensing remains fundamentally restricted by classical sampling theory. Phase-sensitive optical time-domain reflectometers (φ-OTDRs), in particular, encounter inherent trade-offs among spatial resolution, fiber length, and temporal sampling. Unlike the systems dictated by high-speed electronics, the effective sampling rate in φ-OTDRs is bounded by the laser pulse repetition rate (PRR), itself limited by the fiber’s round-trip propagation delay [7]. To transcend the Nyquist sampling ceiling on detectable frequencies, contemporary implementations increasingly leverage methods such as compressed sensing and randomized sampling schemes [8,9,10,11]. However, these techniques are typically realized in the frequency domain, necessitating intricate mathematical frameworks, chiefly the Fourier transform, to resolve spectral content. This frequency-based operation often demands elaborate system architectures, incorporating multiple high-speed components to faithfully capture transient, high-frequency vibrational signatures. The elevated complexity in both signal processing and hardware configuration hinders system scalability, integration, and suitability for real-time deployment.

Conversely, fiber Bragg gratings (FBGs) have emerged as highly sensitive and widely adopted fiber-optic devices for dynamic mechanical stimuli, including vibration-based sensing [12,13,14]. However, the full potential of FBGs in high-frequency vibration sensing is often curtailed by limitations within the optical interrogator hardware. Specifically, low-power and portable FBG interrogators, which are crucial for embedded and field-deployable platforms, are typically restricted by low sampling rates. This challenge becomes even more pronounced when attempting to resolve extremely close frequency components within the same band.

The convergence of machine learning, such as convolutional neural networks (CNNs), long short-term memory (LSTM), deep neural networks (DNNs), linear discriminant analysis (LDA), logistic regression (LR), multi-layer perceptron (MLP), and random forest (RF) [15,16,17,18,19,20], with optical fiber sensing marks a significant paradigm shift. This integration offers a synergistic framework to tackle both longstanding limitations and emerging challenges in the field. Beyond enhancing functional versatility, this fusion elevates optical sensors into a new class of intelligent instruments capable of adaptation, evolution, and the discernment of subtle signal patterns that conventional technique often fails to detect.

**Figure 1:**
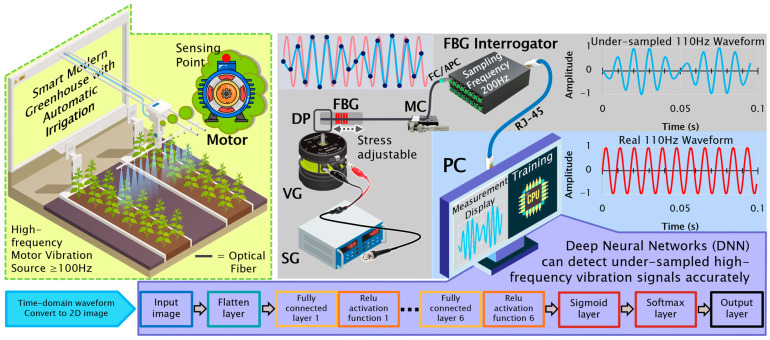
The proposed novel sensing framework that combines an FBG sensor, a compact and low-power, low-sampling-rate FBG interrogator, and an advanced DNN architecture. SG: signal generator, VG: vibration generator, DP: displacement platform, FBG: fiber Bragg grating, MC: micrometer clamp, PC: personal computer.

This study proposes an integrated sensing framework combining an FBG sensor, a low-power, compact FBG interrogator operating at a limited sampling rate (200 Hz), and an advanced DNN architecture, as illustrated in Figure 1. The vibrational excitation was introduced via a vibration generator (VG) driven by a signal generator (SG), mechanically coupled to the proximal end of the optical fiber containing the FBG sensor. A high-precision micrometer clamp (MC) was used to finely tune the axial pre-strain, ensuring optimal spectral sensitivity to spectral shifts. The distal fiber end was connected with a low-sampling-rate FBG interrogator, which digitized the Bragg wavelength modulations and transmitted them to a host computer for acquisition and downstream analysis.

This system is designed to transcend hardware sampling limitations, significantly extending the effective frequency resolution range of fiber-optic vibration sensing. The proposed DNN-based model directly identifies high-frequency patterns from raw time-domain data, transformed into two-dimensional (2D) image representations, without resorting to conventional spectral-domain techniques. The architecture incorporates frequency-sensitive attention mechanisms to extract salient features.

Experimental investigations were conducted across a vibration frequency range of 100–500 Hz under three distinct test conditions. The results demonstrate the model’s efficacy in extending the operational bandwidth of low-power interrogators, achieving a classification accuracy of 94.85%. Extensive validation using the combination of simulation and real experimental data corroborates the superiority of this approach over traditional spectral estimation methods, yielding precise frequency discrimination and robust signal recognition across diverse vibrational regimes.

The key contributions of this study are as follows:

(1) It addresses undersampling limitations by expanding the usable frequency range of low-rate FBG interrogators.
(2) It introduces a compact, low-power optical sensing system suitable for energy-constrained environments.
(3) It enables resolution of extremely close frequency components without reliance on spectral transformations.
(4) It validates real-world deployment feasibility through experimental studies with physical sensors.

Collectively, these results underscore the transformative potential of architectures in advancing the functionality and adaptability of next-generation optical fiber sensing technologies.

## 2. Vibration Signal Sampling Rate

A vibration signal encapsulates the dynamic response of a system to internal or external excitations, typically manifested as oscillatory motion in rotating machinery or structural components. It serves as a dense repository of diagnostic information, reflecting the system’s mechanical health, dynamic behavior, and latent anomalies. Vibration signals are intrinsically multivariate, comprising attributes such as amplitude, frequency, phase, and temporal evolution. Owing to this spectral complexity, continuous monitoring across an expansive frequency spectrum is essential, particularly in the high-frequency domain, where incipient anomalies often precede visible or catastrophic failures. High-frequency vibrations are especially consequential in scenarios involving high-speed machinery, microstructural resonance phenomena, or early-stage mechanical degradation.

In alignment with these considerations, this study identifies and evaluates three prototypical high-frequency cases, each corresponding to a distinct vibratory bandwidth of interest, as summarized in Table 1. The intermediate band spanning 201–299 Hz was intentionally omitted due to the persistently negligible signal amplitudes observed in preliminary assessments, rendering it diagnostically uninformative.

The sampling rate, or sampling frequency, defines the discrete temporal intervals at which a continuous-time signal is probed and transcribed into its digital representation. It constitutes a fundamental parameter in digital signal acquisition, exerting a direct influence on both the temporal resolution and spectral fidelity of the captured signal. Expressed in hertz (Hz), the sampling rate determines the highest recoverable frequency component, as stipulated by the Shannon–Nyquist criterion, and is mathematically defined as follows [21,22]:

(1) $$f_{s}\geq 2f_{max}$$

(2) $$f_{us}=\left|f-kf_{s}\right|$$

where $f_{s}$ represents the sampling frequency, and $f_{max}$ is the maximum frequency. If the signal is sampled at a rate lower than $2f_{max}$, undersampling occurs. $f_{us}$ is the undersampled frequency, f is the measured frequency, and k is the nearest integer such that $f_{us}$ falls within the Nyquist range.

**Figure 2:**
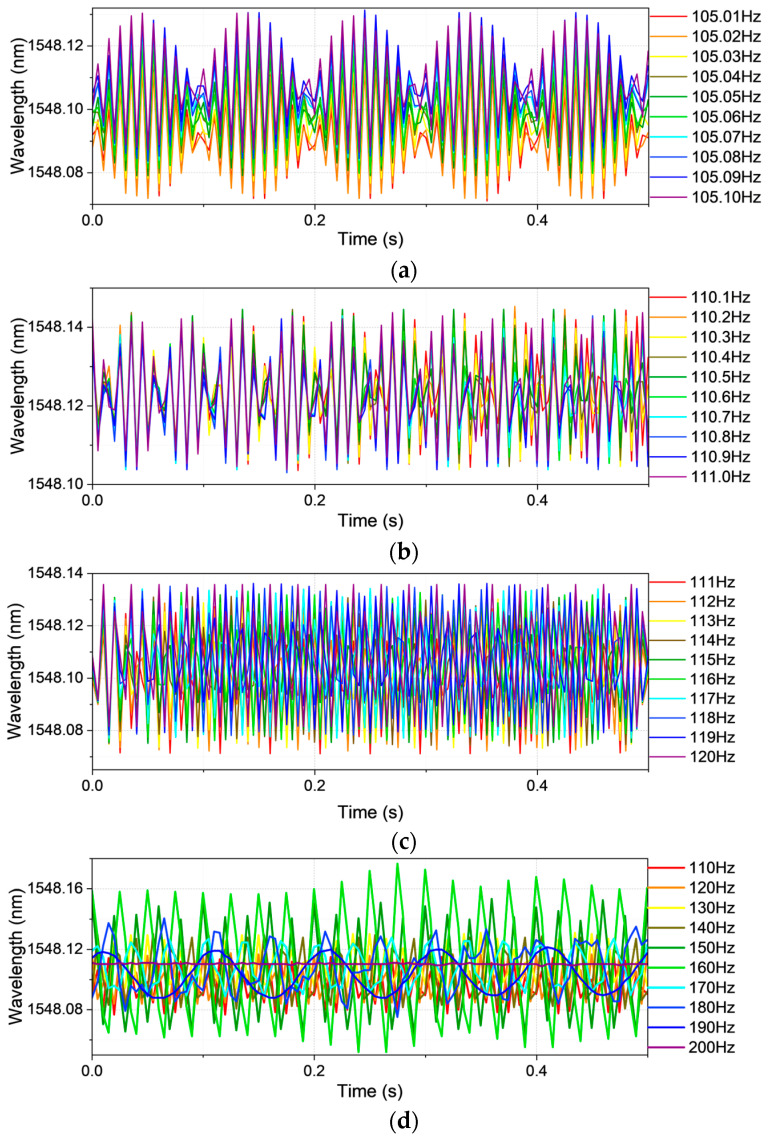
The time-domain vibration waveform for Case 1: (**a**) frequency 105.01–105.10 Hz with data spacing 0.01 Hz; (**b**) frequency 110.1–111.0 Hz with data spacing 0.1 Hz; (**c**) frequency 111–120 Hz with data spacing 1 Hz; (**d**) frequency 110–200 Hz with data spacing 10 Hz.

The undersampled time-domain waveforms corresponding to vibration frequency Case 1 are depicted in Figure 2, illustrating the raw experimental signals acquired across varying frequency intervals. Figure 2a presents a narrowly constrained frequency band of 105.01–105.10 Hz with an ultra-fine resolution of 0.01 Hz. Despite this high granularity, the waveform reveals no coherent structure reflective of the true vibrational dynamics, indicating a pronounced degradation in spectral fidelity—a consequence of Shannon–Nyquist sampling limitations. This spectral distortion remains evident across broader frequency spans, as illustrated in Figure 2b–d, corresponding to data spacings of 0.1 Hz, 1 Hz, and 10 Hz, respectively.

Beyond theoretical conformity, the sampling rate encapsulates a triad of operational imperatives: (1) temporal resolution, dictating the granularity of captured dynamics; (2) bandwidth compatibility, defining the upper bound of observable frequency content; and (3) computational burden, wherein elevated sampling rates escalate data volume and processing requirements. In the proposed system, the fiber Bragg grating (FBG) interrogator adopts a uniform sampling regime—computationally efficient but inherently suboptimal for discerning narrowly spaced spectral components. This rigidity, imposed by fixed temporal intervals, induces spectral leakage and modal ambiguity, particularly within congested high-frequency domains.

**Figure 3:**
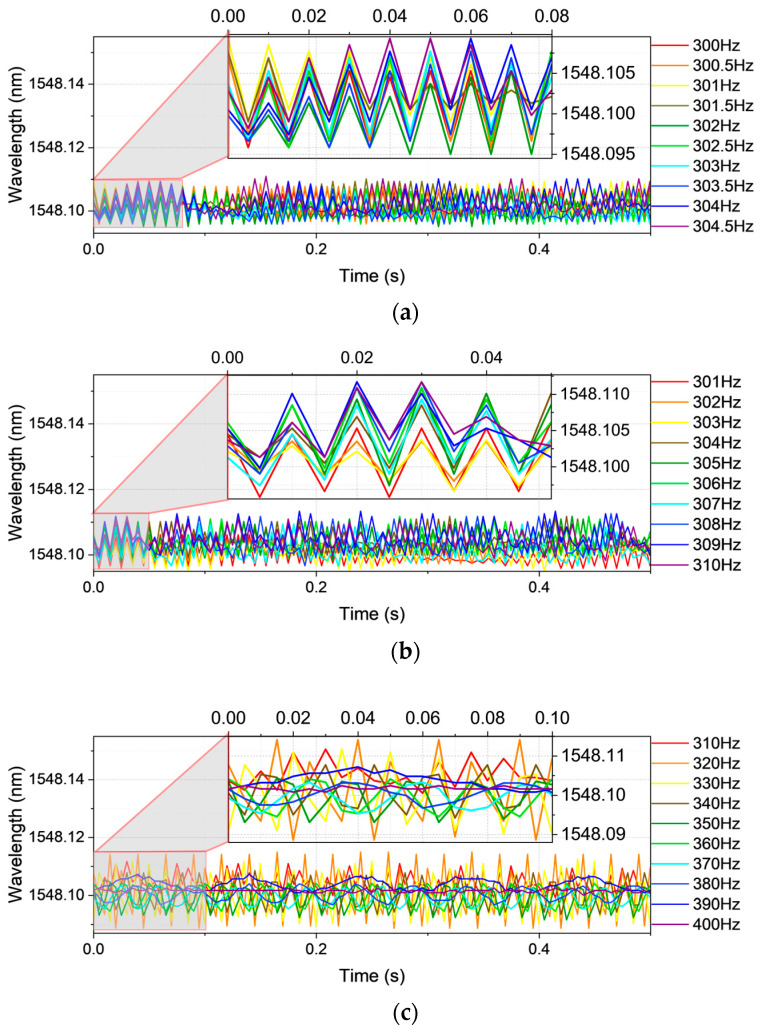
The time-domain vibration waveform for Case 2: (**a**) frequency 300.0–304.5 Hz with data spacing 0.5 Hz; (**b**) frequency 301–310 Hz with data spacing 1 Hz; (**c**) frequency 310–400 Hz with data spacing 10 Hz.

Vibration frequency Case 2, delineated in Figure 3, further corroborates this constraint. The waveforms span 300.0–304.5 Hz at 0.5 Hz spacing (Figure 3a), 301–310 Hz at 1 Hz (Figure 3b), and 310–400 Hz at 10 Hz (Figure 3c). In this case, the frequency content surpasses the intrinsic sampling threshold of the FBG interrogator (200 Hz), leading to spectral attenuation and diminished power magnitude relative to Case 1 (100–200 Hz). This misalignment between signal content and sampling capacity erodes temporal fidelity, rendering the time-domain representation increasingly indistinct.

**Figure 4:**
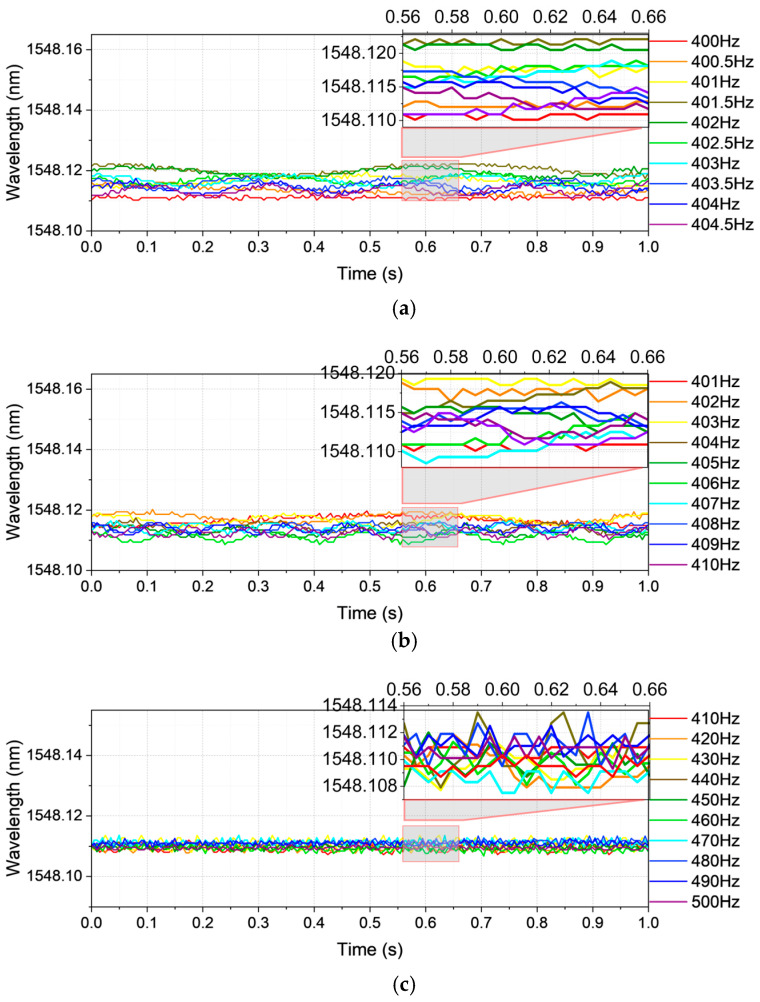
The time-domain vibration waveform for Case 3: (**a**) frequency 400.0–404.5 Hz with data spacing 0.5 Hz; (**b**) frequency 401–410 Hz with data spacing 1 Hz; (**c**) frequency 410–500 Hz with data spacing 10 Hz.

The final investigation, vibration frequency Case 3, shown in Figure 4, examines bands of 400.0–405.0 Hz at 0.5 Hz spacing (Figure 4a), 401–410 Hz at 1 Hz (Figure 4b), and 410–500 Hz at 10 Hz (Figure 4c). Here, again, the waveforms exhibit weakened structural definition, making the constituent frequencies practically indiscernible. This recurring phenomenon underscores the deleterious impact of undersampling on high-frequency signal recognition.

Collectively, these results affirm that the fidelity of vibration signal acquisition in fiber-optic sensing is intrinsically coupled to the sampling rate—a critical interface between analog vibratory phenomena and their digital representation. Suboptimal sampling precipitates spectral aliasing, thereby obscuring diagnostically salient features such as resonance shifts, damping signatures, or transient anomalies [23,24,25,26]. Accordingly, beyond nominal compliance with the Shannon–Nyquist condition, the sampling frequency must be judiciously tailored to both the system’s bandwidth and the spectral characteristics of the monitored environment. These findings reinforce the necessity for intelligent signal recognition paradigms capable of extracting high-fidelity spectral insights from alias-contaminated, undersampled time-series data.

## 3. The Proposed Systems

The proposed sensing framework embodies a compact, energy-conscious architecture that seamlessly integrates photonic and neural modules for high-fidelity physical interaction monitoring. At its nucleus resides a fiber Bragg grating (FBG) sensor (centered at 1548 nm), meticulously affixed via single-mode optical fiber and secured by a micrometer-scale clamp (MC), thereby ensuring mechanical stability and consistent strain responsivity. Strain-induced spectral perturbations are transduced by a commercial, multi-channel FBG interrogator operating across the 1528–1568 nm wavelength domain. Tailored for constrained environments, this interrogator operates at intentionally reduced sampling frequencies to balance signal discernibility and energy economy, consuming merely 30 W under a 5 VDC/6 A input and functioning reliably within a –15 to 65 °C operational envelope. The resulting undersampled vibration waveform, contrasted with its ground-truth waveform, is depicted in Figure 1, emphasizing the disparity between the aliased raw input and the recognized signal derived via deep neural network (DNN) inference.

Vibrational stimuli are imparted using a signal generator (SG) connected with a vibration generator (VG), which excites a displacement platform (DP) to emulate diverse mechanical excitations. The resultant strain-induced wavelength shifts in the FBG are interrogated, digitized, and subsequently relayed to a host computer (PC) for visualization, processing, and model training.

A bespoke DNN was meticulously architected to classify high-dimensional spectral representations and to infer latent harmonic structures embedded in undersampled temporal signals. Eschewing convolutional paradigms, the model adopts a fully connected architecture to exploit dense, layer-wise representations. The input comprises RGB images of dimension 224 × 224 × 3, which are reshaped into a unidimensional vector of 50,176 features via a Flatten operation. This transformation preserves all input variability while allowing for direct interaction between distal pixels—an essential feature for uncovering dispersed high-frequency components whose patterns may not be spatially localized.

**Figure 5:**
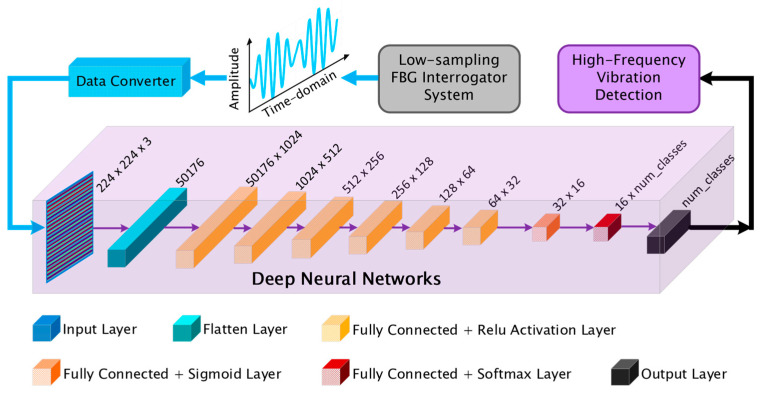
The structure of the proposed DNN model.

The flattened vector propagates through a deep sequence of ten fully connected (FC) layers, as illustrated in Figure 5. These layers progressively reduce the feature dimensionality, 1024 → 512 → 256 → 128 → 64 → 32 → 16, culminating in an output layer parameterized by the number of target classes. Each FC layer performs a full affine transformation of its input space, enabling the model to learn intricate inter-feature dependencies and to approximate the nonlinear mappings intrinsic to aliased signal manifolds. The elevated parameter count (53,406,368 trainable weights) equips the model with substantial representational capacity, critical for disentangling genuine spectral content from sparsely sampled observations.

To mitigate overfitting—an endemic challenge in overparameterized architectures—the network enforces structural parsimony via progressive dimensionality compression. This compressive topology compels the abstraction of only the most discriminative features across depth.

Nonlinearity is instilled through ReLU activations in the first six hidden layers. ReLU imparts sparsity by attenuating subthreshold activations, thereby amplifying dominant signal modulations commonly associated with aliased harmonics. This is followed by a sigmoid activation in the penultimate layer, which compresses activations into a bounded range, acting as a gating mechanism to stabilize the feature space before classification. The final softmax layer normalizes the class logits into probability distributions, enhancing interpretability and ensuring compatibility with the CrossEntropyLoss function used during training. Training optimization was conducted using the Adam optimizer with a constant learning rate of 0.001, balancing adaptive momentum updates and robust convergence in high-dimensional parameter spaces.

The model was trained for 100 epochs with a batch size of 30, utilizing shuffled data to prevent temporal dependencies. Inputs were standardized via zero-centered normalization (mean = 0.5, std = 0.5) across all channels, ensuring consistent scaling and improved numerical stability. The training pipeline was deployed on a GPU to expedite computation. No explicit regularization (e.g., dropout or weight decay) was incorporated, thereby preserving architectural transparency and enabling full exploitation of the network’s representational capacity. Despite the absence of convolutional operations, the model demonstrated remarkable aptitude in learning implicit frequency-domain characteristics through its deep, dense architecture. In particular, the use of fully connected layers facilitated the capture of global signal interactions, critical when dealing with undersampled inputs where local patterns alone may prove insufficient.

The trained model was stored using PyTorch’s .pth format and evaluated via forward-pass on preprocessed image tensors. Training was executed on a system with a 13th-gen Intel Core i9, 32 GB RAM, and an NVIDIA RTX 3090 GPU (10 GB VRAM), enabling efficient GPU-accelerated computation.

**Figure 6:**
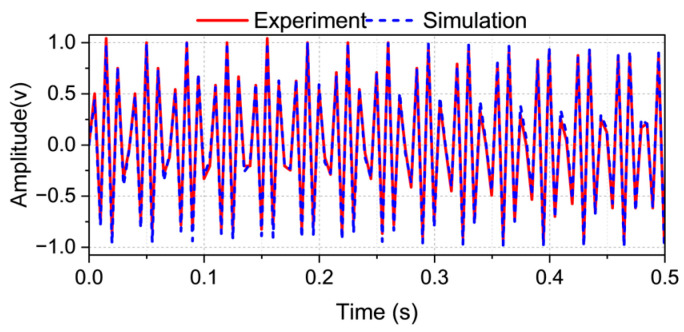
The comparison of the experiment and simulation waveforms at a frequency of 114.5 Hz.

The dataset used to train and validate this architecture comprised a hybrid corpus of empirical sensor data and synthetic simulations, enabling the network to generalize across measurement noise and signal degradation. A comparative visualization between experimental and simulated results is presented in Figure 6, illustrating the model’s capacity to generalize across data modalities.

## 4. Results and Discussion

**Figure 7:**
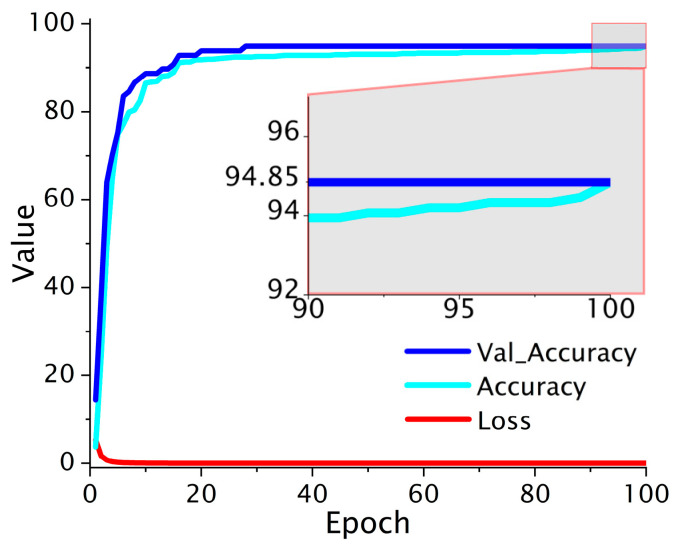
The DNN loss-accuracy result.

The performance trajectory of the proposed deep learning model reveals both expedited convergence and long-term stability, as substantiated by its validation accuracy and loss dynamics. Notably, the model attains a stable validation accuracy of 94.85% from epoch 33 onward, maintaining this high precision through epoch 100, thereby demonstrating both convergence and generalization capability as shown in Figure 7. The loss curve reaches its global minimum by epoch 7, indicating early stabilization of the network’s internal feature abstraction and suggesting a well-conditioned optimization landscape.

**Figure 8:**
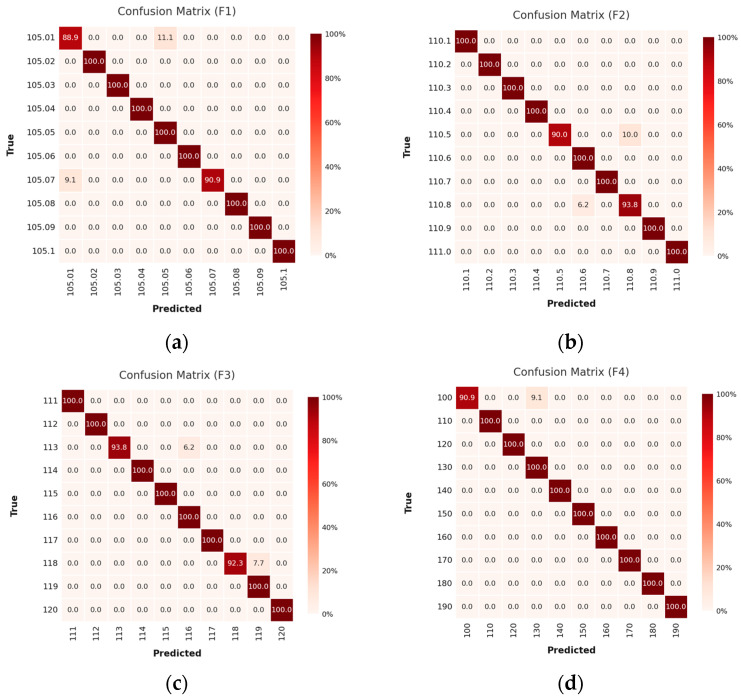
The confusion matrix results of Case 1: (**a**) frequency 105.01–105.10 Hz with data spacing 0.01 Hz; (**b**) frequency 110.1–111.0 Hz with data spacing 0.1 Hz; (**c**) frequency 110–120 Hz with data spacing 1 Hz; (**d**) frequency 110–190 Hz with data spacing 10.

The framework’s efficacy is further corroborated through confusion matrices derived from a comprehensive suite of high-resolution frequency classification tasks, wherein initial and terminal frequencies were systematically varied to evaluate the model’s adaptability across disparate high-frequency regimes. In vibration frequency Case 1 (Figure 8), the model exhibits remarkable acuity in discriminating narrowly spaced signals, 105.01–105.10 Hz at ultra-fine 0.01 Hz increments (Figure 8a) and 110.1–111.0 Hz at 0.1 Hz (Figure 8b), as well as broader spans such as 111–120 Hz at 1 Hz and 100–190 Hz at 10 Hz intervals (Figure 8c,d, respectively).

**Figure 9:**
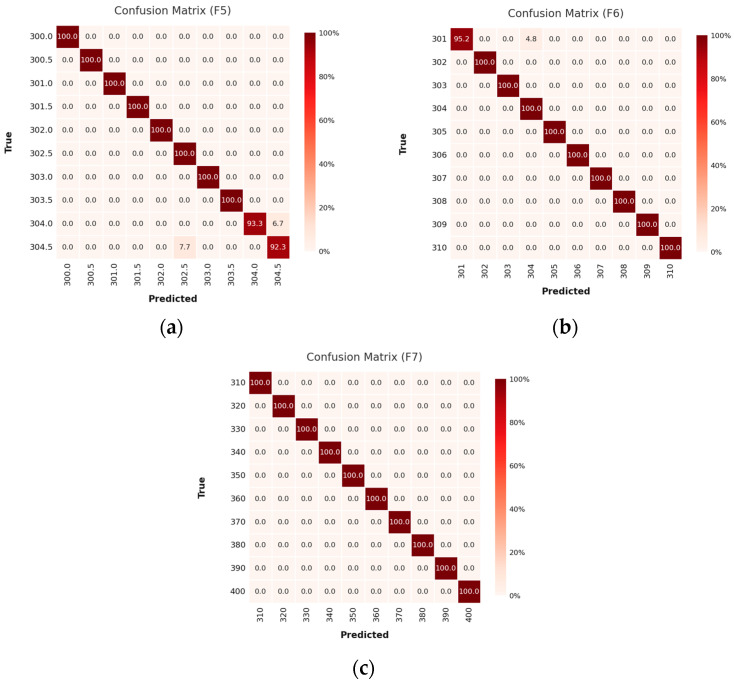
The confusion matrix results of Case 2: (**a**) frequency 300.0–304.5 Hz with data spacing 0.5 Hz; (**b**) frequency 301–310 Hz with data spacing 1 Hz; (**c**) frequency 310–400 Hz with data spacing 10.

The confusion matrices display sharply delineated classification boundaries and a negligible misclassification rate, affirming the model’s precision in resolving subtle spectral variations. This pattern of robust performance persists in vibration frequency Case 2 (Figure 9), where the model accurately categorizes signals across the 300.0–304.5 Hz range at 0.5 Hz spacing (Figure 9a), 301–310 Hz at 1 Hz (Figure 9b), and 310–400 Hz at 10 Hz (Figure 9c). The model sustains high fidelity even under coarser frequency discretization, reflecting its scalability across multi-resolution domains.

**Figure 10:**
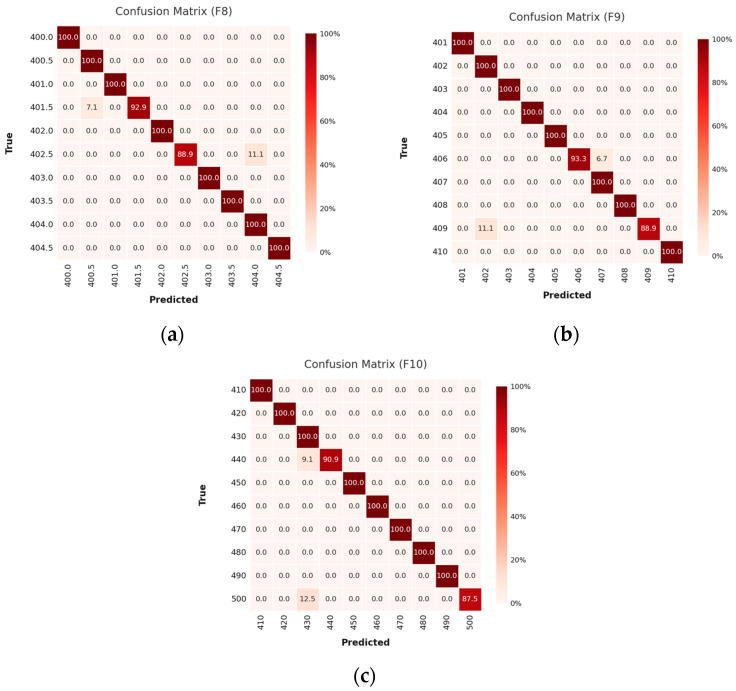
The confusion matrix results of Case 3: (**a**) frequency 400.0–404.5 Hz with data spacing 0.5 Hz; (**b**) frequency 401–410 Hz with data spacing 1 Hz; (**c**) frequency 410–500 Hz with data spacing 10.

In vibration frequency Case 3 (Figure 10), the network maintains its discriminative acuity across increasingly challenging spans: 400.0–404.5 Hz at 0.5 Hz (Figure 10a), 401–410 Hz at 1 Hz (Figure 10b), and 410–500 Hz at 10 Hz spacing (Figure 10c). In all instances, the model maps input signals to their correct frequency classes with precision, regardless of frequency magnitude or resolution granularity.

Collectively, these empirical results underscore the proposed model’s robust spectral resolution and its consistent capacity for fine-grained classification of high-frequency vibrations. Achieving this level of accuracy using raw time-domain signals from low-sampling-rate optical sensors highlights the system’s potential as a compact, low-power, and high-resolution frequency recognition solution for next-generation photonic sensing platforms.

## 5. Conclusions

This research introduces a novel deep neural network (DNN) architecture that transcends conventional sampling limitations in high-frequency vibration recognition using fiber Bragg grating (FBG) sensors, achieving a classification accuracy of 94.85% with signals acquired from low-power, low-sampling-rate FBG interrogators. The model proficiently discerns extremely closed and high-frequency vibratory components without the aid of traditional signal processing techniques or spectral enhancement algorithms.

Validated through rigorous evaluation on a composite dataset comprising both simulated and empirical measurements, the proposed framework demonstrates robust recognition performance across the 100–500 Hz frequency spectrum. This capability highlights its effectiveness in recovering spectral information typically lost due to undersampling, thereby extending the functional bandwidth of resource-constrained optical sensing platforms.

The study presents a compact, power-efficient, and intelligent vibration sensing paradigm, ideally suited for deployment in spatially constrained or remote environments where conventional high-speed data acquisition is impractical.

## Figures and Tables

**Table 1:** Table of case study.

Cases	Frequency Range
Case 1	100–200 Hz
Case 2	300–400 Hz
Case 3	400–500 Hz

## Data Availability

The data presented in this study are available in this article.
